# Maturity Ogives for South Pacific Albacore Tuna (*Thunnus alalunga*) That Account for Spatial and Seasonal Variation in the Distributions of Mature and Immature Fish

**DOI:** 10.1371/journal.pone.0083017

**Published:** 2014-01-08

**Authors:** Jessica H. Farley, Simon D. Hoyle, J. Paige Eveson, Ashley J. Williams, Campbell R. Davies, Simon J. Nicol

**Affiliations:** 1 Wealth from Oceans Flagship, CSIRO Marine and Atmospheric Research, Hobart, Tasmania, Australia; 2 Oceanic Fisheries Programme, Secretariat of the Pacific Community, Noumea, New Caledonia; Technical University of Denmark, Denmark

## Abstract

Length and age at maturity are important life history parameters for estimating spawning stock biomass and reproductive potential of fish stocks. Bias in estimates of size and age at maturity can arise when disparate distributions of mature and immature fish within a population are not accounted for in the analysis. Here we investigate the spatial and temporal variability in observed size and age at maturity of female albacore tuna, *Thunnus alalunga*, using samples collected across the South Pacific. Maturity status was identified using consistent histological criteria that were precise enough to allow for mature but regenerating females to be distinguished from immature females during the non-spawning season, permitting year-round sampling for maturity estimation in albacore. Using generalised linear mixed models, we found that the proportion of mature females at length varied significantly with latitude and time of year. Specifically, females at northern latitudes (∼10–20°S, where spawning occurs) were mature at significantly smaller lengths and ages than females at southern latitudes (∼20–40°S), particularly during the spawning season (October–March). This variation was due to different geographic distributions of mature and immature fish during the year. We present a method for estimating an unbiased maturity ogive that takes into account the latitudinal variation in proportion mature at length during a given season (spawning or non-spawning). Applying this method to albacore samples from the western region of the South Pacific gave a predicted length at 50% mature of ∼87 cm fork length (4.5 years).

## Introduction

Length and age at maturity are important life history parameters that are often used when assessing stock status and estimating spawning stock biomass or reproductive potential. They are typically obtained by examining ovaries using macroscopic or histological techniques to determine maturity status of individuals and then applying statistical models to determine the proportion mature as a function of length or age [1]. It is generally acknowledged that these maturity ogives can vary spatially and temporally, and this variability needs to be understood and accounted for when used in assessment models [2]. Spatial variability in maturity can be due to variation in growth rates of a species, providing evidence for stock structure within a population [3]. However, variability can also result from disparate distributions of mature and immature fish, which may bias the estimated maturity ogive depending on the distribution of the sampling effort. For instance, estimates of length at 50% maturity will be lower for fish sampled in spawning areas than for fish sampled elsewhere if mature fish migrate to spawning areas and immature fish do not. In such cases, maturity ogives based on samples from spawning areas will not represent the whole population [4]. Similarly, if the seasonal movements of mature and immature fish differ, then the timing of the sampling will affect the estimated maturity ogives. Hence, it is important to account for potential spatial and temporal variability in maturity both when designing the sampling program and when estimating a population ogive from the resulting maturity data.

Albacore tuna, *Thunnus alalunga*, are one of only four tuna species that are considered truly migratory, and move seasonally to specific feeding and spawning areas (along with southern bluefin *Thunnus maccoyii*, Pacific bluefin *T. orientalis* and Atlantic bluefin *T. thynnus* tunas) [5]. In the South Pacific, albacore spawn in the western and central region between 10°S and 25°S and to ∼140°W during a relatively protracted spring/summer season [6]. Juveniles at age 1 year are caught south of the spawning latitudes in surface waters around New Zealand at approximately 40°S and in the subtropical convergence zone. Juveniles gradually disperse north as they grow, returning to latitudes north of 30°S only as adults [6], [7], where they are caught by longline fishing gear. This dispersal pattern results in a distinct size gradient by latitude with the average size of fish caught increasing with decreasing latitude. Catch rate data suggest that adult albacore also make seasonal north-south migrations possibly following specific temperature isotherms [7]. Given the spatial stratification of adults and juveniles with latitude, and seasonal movements, estimating the proportion of the population mature with size or age requires a large-scale sampling program that encompasses the full geographic range of the stock, with good representation across seasons, and an adequate number of samples from the size range over which the transition to maturity occurs [8]. Appropriate histological criteria to identify the maturity status of individuals and a method to account for the spatial and temporal distributions of adults/juveniles are also required [5], [8].

Regional albacore stock assessments (e.g. for the Atlantic, Indian and North Pacific) use maturity ogives to generate the ‘spawning stock’ for the stock recruitment relationship, but do not take spatial and temporal variation in maturity into account. In the South Pacific, there are no quantitative estimates of size or age at maturity of albacore [5]. Since 2008, the South Pacific regional stock assessment has used the proportion of females mature at age, based on the assumption that 50% maturity is reached at 85 cm fork length (*FL*) [9], [10], [11], as a component to calculate relative reproductive potential. Although the assessment of South Pacific albacore indicates that the stock is not overfished and that overfishing is not occurring [11], a better understanding of maturity, migration and population structure are important to better inform the assessment model structure. Even a small difference in age at 50% maturity relative to age at 50% selection can have a considerable effect on estimates of maximum sustainable yield for albacore [12].

The objectives of the current study were to examine the spatial and seasonal variability in maturity at length and age for female albacore in the South Pacific Ocean, and propose a statistical method to obtain an average maturity ogive for the population when spatial and temporal variability exists. The relative abundance by sex data needed to produce an average maturity ogive for the entire South Pacific were not available; however, the method was applied to maturity data for the component of the stock harvested by Australia's Eastern Tuna and Billfish Fishery (ETBF).

## Methods

### Ethical statement

Ethical approval was not required for this study, as all fish were collected as part of routine fishing procedures. No samples were collected by the authors. All samples in this study originated from commercial or recreational fisheries (New Zealand commercial Albacore Fishery, Western Central Pacific Ocean commercial longline fishery, and Australian commercial ETBF and recreational fishery) and were already dead when provided to the sampler. Fish were sacrificed by the commercial or recreational fisher at sea using standard fisheries practices (most fish were dead when landed). Permission was granted to use samples from all fish. All samples were donated.

No field permits were required to collect any samples from any location, as all samples originated from commercial and recreational catch. Albacore tuna are not a protected species in any ocean.

### Data collection

**Figure 1:**
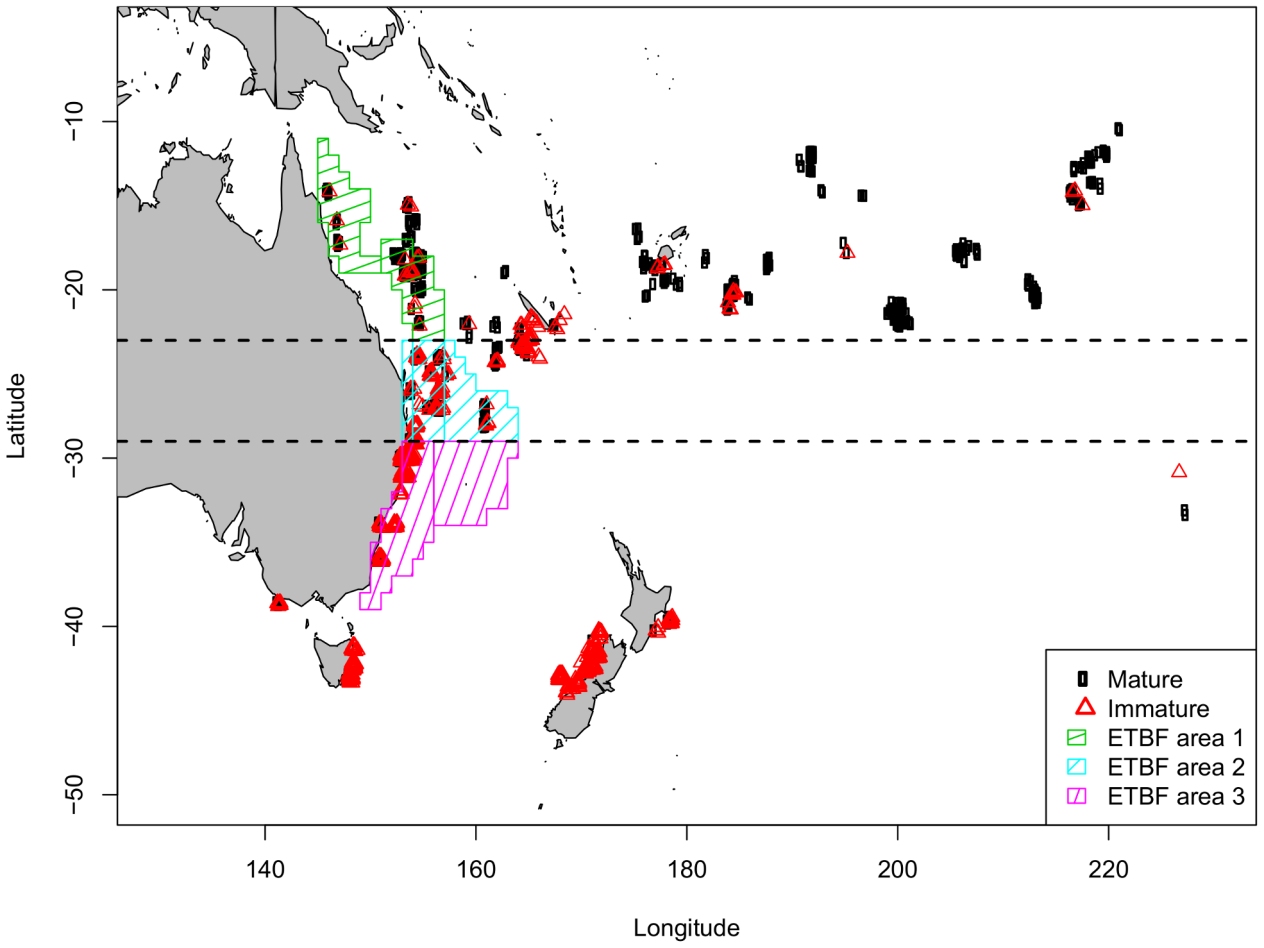
Sample locations of mature and immature female albacore in the South Pacific Ocean. The horizontal dashed lines define the latitudinal bands used in the model with a discrete latitude covariate (<23°S, 23–29°S, >29°S). Also shown are the areas for which standardized CPUE estimates for albacore tuna in Australia's Eastern Tuna and Billfish Fishery (ETBF) have been produced and the three latitudinal areas into which they were divided for the maturity analysis. Longitude is shown as degrees east; i.e., 160°W is shown as 200°E.

A large biological sampling program for albacore tuna was undertaken across the southwest Pacific Ocean between 2006 and 2011 [6]. Ovaries and otoliths were collected from fish caught across a broad geographic area with almost the entire longitudinal range of the species sampled, including a broad latitudinal range west of 175°E (Fig. 1). The majority of samples were collected from fish caught in commercial troll and longline fisheries in Australia, New Zealand, New Caledonia, Fiji, Tonga, American Samoa, Cook Islands, French Polynesia, and in a region south of the Pitcairn Islands. Samples from Australia were collected in port from fish caught by the domestic longline fishery along almost the entire mainland coast between 14°S and 37°S. Additional samples were collected from small fish caught by recreational fishers in southern Australia between 37°S and 44°S. Samples from New Zealand were collected either in port from the domestic troll fishery or at sea during chartered tagging operations. Samples collected from all other regions were collected either by observers on longline fishing vessels or directly by the fishing crew of longline fishing vessels.

**Figure 2:**
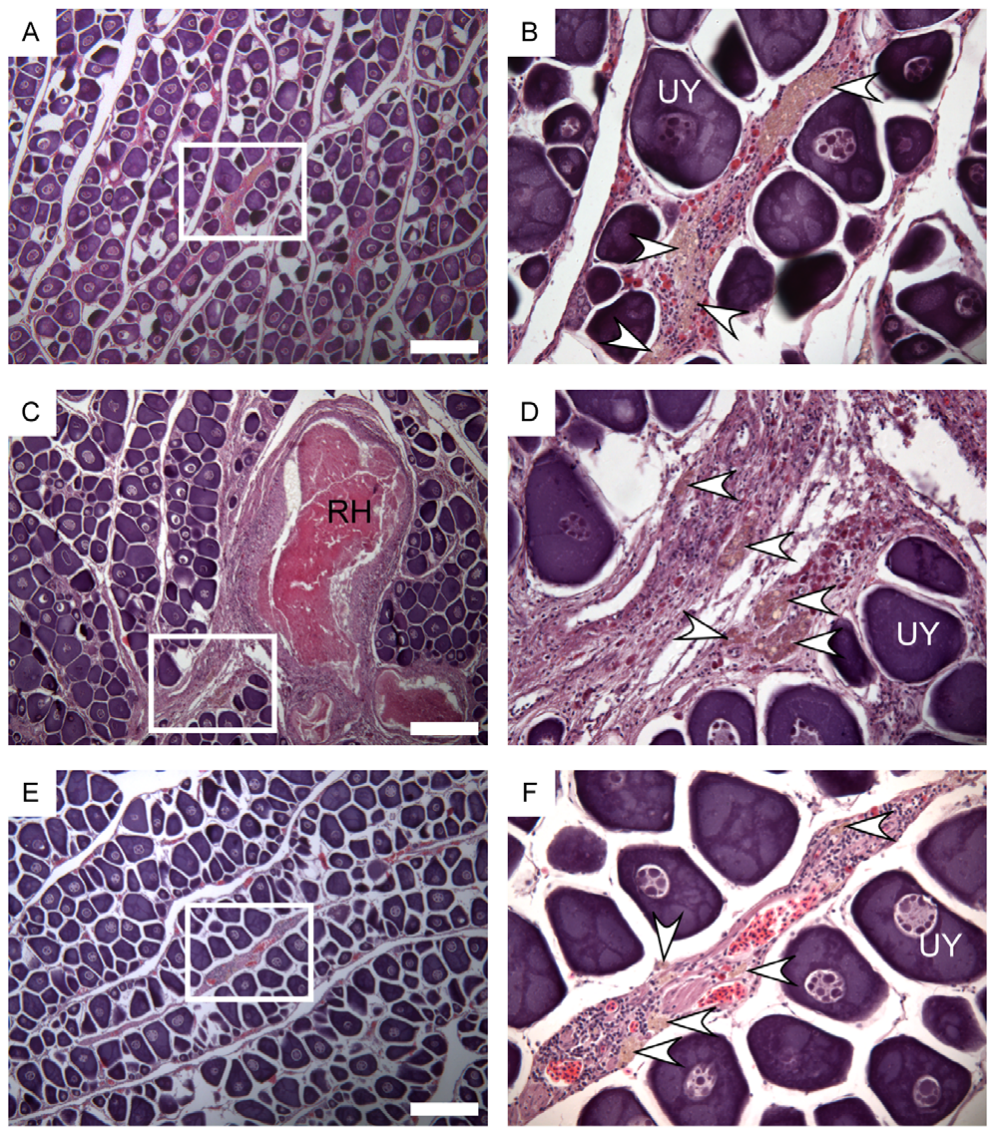
Histological sections of ovaries of mature females classed as regenerating. White box in left panels are shown under higher magnification in right panels. (A, B) 92 cm *FL* female sampled in June 2010. (C, D) 88 cm *FL* female sampled in August 2010. (E, F) 100 cm FL female sampled in September 2010. Arrows = late stage atresia (gamma/delta; brown bodies), RH = residual (unovulated) hydrated oocyte, UY = unyolked oocytes. The white scale bars is 200 µm.

The *FL* of fish sampled and the reproductive development class (phase) of ovaries were obtained directly from [6]. In that study, the ovaries of all females ≥70 cm *FL* (n = 1219) were selected for histological analysis as these were larger than the expected minimum size at maturity. Females <70 cm *FL* were classed as immature based on macroscopic examination of the ovaries. Ovaries selected for histology were classified into 7 development classes based on (1) the most advanced group of oocytes, (2) the presence/absence of postovulatory follicles, (3) the presence/absence of alpha or beta stage atresia of yolked oocytes, and (4) presence/absence of maturity markers (Table 1). Females were classified as mature if their ovaries contained yolked oocytes (advanced, migratory nucleus or hydrated), atresia of yolked oocytes (alpha or beta stage) or maturity markers (Table 1). Maturity markers were either residual (unovulated) hydrated oocytes or very late stages of atresia of yolked oocytes (gamma or delta; see [13], [14]). The late stages of atresia are often referred to as brown or orange bodies ([6], [13], [15]) as they appear as small yellow/brown/orange coloured granular structures within the ovary tissue (Fig. 2). Their presence is evidence of previous development of yolked oocytes and reproductive activity [13]. Mature females were further classed as active (spawning capable or spawning) or inactive (regressing, regressed or regenerating) (Table 1). Immature fish were characterised by the presence of unyolked or early yolked oocytes as the most advanced stage present in the ovary, and no atresia or maturity markers [6] (Table 1; Fig. 2).

**Table 1:** Number of South Pacific albacore by histological classification.

Class	Maturity status	Activity	Development class	MAGO and POF stage	α and β atresia of yolked oocytes	Maturity markers	Count
1	Immature	Inactive	Immature	Unyolked, no POFs	Absent	None	667
2	Immature	Inactive	Developing	Early yolked, no POFs	Absent	None	17
3	Mature	Active	Spawning capable	Advanced yolked, no POFs	<50% α atresia, β atresia may be present	May be present	67
4	Mature	Active	Spawning	Migratory nucleus or hydrated and/or POF's	<50% α atresia, β atresia may be present	May be present	356
5	Mature	Inactive	Regressing - potentially reproductive	Advanced yolked, no POFs	≥50% α atresia, β atresia present	May be present	9
6a	Mature	Inactive	Regressed 1	Unyolked or early yolked, no POFs	100% α atresia, β atresia may be present	May be present	27
6b	Mature	Inactive	Regressed 2	Unyolked or early yolked, no POFs	No α atresia, β atresia present	May be present	40
7	Mature	Inactive	Regenerating	Unyolked or early yolked, no POFs	Absent	Present	313

MAGO = most advanced group of oocytes, POF = postovulatory follicle. Maturity markers include gamma and delta stages of atresia (brown bodies) and/or residual (unovulated) hydrated oocytes.

Estimates of age were obtained from a previous study [16] from counts of annual increments in otoliths for 951 females sampled between January 2009 and December 2010. Otoliths were selected for age estimation based on sampling location and *FL* with the aim of estimating age for the full size range of females caught across a broad area of the southwest Pacific Ocean.

### Spatial and temporal variation in maturity

We used generalized linear mixed models, with the lme4 package [17] in R version 2.13.2 [18] to statistically examine variation in length and age at maturity of albacore with latitude, longitude and time of year. Maturity state was treated as a binomial response variable with logit link function and modelled as a function of either age or *FL*, with spatial and temporal covariates latitude, longitude and season. Season was modelled as a categorical variable with either two levels (Oct–Mar and Apr–Sep) or four levels (Jan–Mar, Apr–Jun, Jul–Sep and Oct–Dec). Age, *FL*, latitude and longitude were modelled as linear variables or as cubic splines with nodes at equally-spaced quantiles of the data. AIC_c_ (as described below) was used to estimate the most appropriate number of nodes (*k*), or degrees of freedom (df = *k*−1). Fishing set was included in the model as a random effect term, because multiple individual fish were often sampled at the same time from a single location and, therefore, not all samples were independent.

We used an information theoretic, multi-model inference approach to compare alternative models and investigate which covariates and interaction terms should be included. We evaluated the relative support for each model using Akaike's Information Criterion for small sample sizes (AIC_c_; [19]). Models with an AIC_c_ value within two of that calculated for the best approximating model (lowest AIC_c_) were considered to describe the data equivalently well [19]. The Akaike weight, *w_i_* [19], of each model *i* was calculated to quantify the plausibility of each model, given the data and the set of models, using:
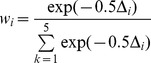
where Δ*_i_* = AIC_c,min_−AIC_c,*i*_. The Akaike weight represents the weight of evidence in favour of model *i* being the best model in the set.

We compared the relative explanatory power of *FL* and age as predictors of maturity by taking the subset of data for which ages had been estimated and comparing model fits using AIC_c_.

### Maturity ogive estimation

Analyses done in the previous section showed that maturity at length for albacore in the South Pacific varies by latitude and season, with an increasing probability of a female of a given length being mature at higher latitudes in the spring/summer spawning season (see Results). If we estimate a single maturity ogive by pooling samples across space and time, then it will be biased unless sampling was in proportion to abundance. As such, we developed a method to calculate a single ‘weighted’ maturity ogive for each season that takes into account spatial variability in maturity and relative abundance.

The best fitting model from the previous section (see Table 2, Model 1) included *FL* and latitude as continuous cubic splines with 2 df, season as a factor with two levels (Oct–Mar and Apr–Sep), and an interaction between season and latitude. To estimate a single weighted maturity ogive, we need estimates of the probability of maturity for discrete areas. Therefore, we refit this model including latitude as a discrete variable with three levels (see Fig. 1) rather than as a cubic spline. As discussed below, the latitudinal divisions were chosen to correspond to areas for which we have relative abundance estimates.

**Table 2:** Comparison of all length-based models with AICc weights greater than 1%.

Model	Len df	Lat df	Lon df	Ssn	Lat * Ssn	Lon* Ssn	Δ AIC_c_	AIC_c_ weights
1	2	2		2	Y		0	46.1%
2	2	2	1	2	Y		0.66	33.2%
3	2	2	1	2	Y	Y	2.62	12.4%
4	1	2		2	Y		5.29	3.3%
5	2	2		4	Y		6.07	2.2%
6	2	2	1	4	Y		7.19	1.3%

The Len df, Lat df, and Lon df columns specify the number of degrees of freedom (df) in the cubic spline used to define the relationship of the parameters length, latitude, and longitude with maturity. Where no df are given, the parameter was not included in the model. The Ssn column specifies the number of levels included in the model for season, either 4 (Jan–Mar, Apr–Jun, Jul–Sep, Oct–Dec) or 2 (Oct–Mar, Apr–Sep). A “Y” in the column for Lat*Ssn indicates that an interaction term between latitude and season was included; similarly for Lon*Ssn.

Using this model, we predicted the proportion of females of *FL l* that are mature in season *s* in each latitudinal area *a*, denoted by 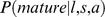. Then, to estimate the proportion of females of *FL l* that are mature across all areas in season *s*, 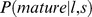, we calculated a weighted average of the area-specific predicted proportions mature, where the weights are the estimated proportions of female abundance by length in each latitudinal area in season *s*, 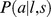. That is:
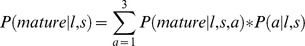
where
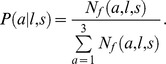


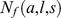
 is the number of females of *FL l* in area *a* in season *s*. We estimate 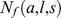 as follows:
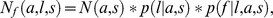
where 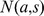 is number of fish (male and female) in latitudinal area 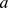 in season *s*, 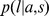 is the proportion of fish in area 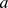 in season *s* of *FL* 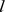, and 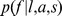 is the proportion of fish of *FL* 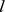 in area 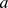 in season *s* that are female.

Ideally, 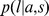 would be estimated using length samples from the catch within each area and season, assuming the samples are representative of the population or else taking into account selectivity. Length data for albacore catches across the South Pacific were not readily available, so instead we used the length distribution of all fish sampled for the study by [6] (males and females combined). We used 5 cm length classes (46–50 cm, 51–55 cm, etc.) to obtain adequate sample sizes within each length class, latitudinal area and season. We estimated the proportion of females within each length class, latitudinal area and season, 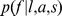, from the same data, assuming that the sex ratio at length in our sample is representative of the population.

Unfortunately, estimates of 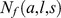 cannot be derived for albacore across the entire South Pacific because abundance estimates by area, season and sex are not available. However, we do have the necessary information available for the Australian ETBF region. Thus, for illustrative purposes, we derived a weighted maturity ogive in each season for female albacore in the ETBF, rather than for the whole South Pacific. 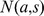 was obtained using estimates of the relative number of fish by area and season from standardised commercial longline CPUE for the ETBF [20]. Only relative abundance estimates are necessary since the weights are proportions scaled to sum to 1. Seven area definitions were used in the CPUE standardisation for the ETBF, but for the purpose of estimating a single maturity ogive, we combined CPUE areas within each of the three latitudinal bands (Fig. 1). Note that our choice of how to define discrete latitude levels in the model was made with consideration for these CPUE areas.

It is important to have estimates not only of the mean proportion mature at length, but also the variance. We calculated the variance of the predicted proportion of mature females by length for the ETBF in season *s* using:
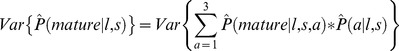



To make the calculation more tractable, we assume that 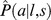 is known accurately, in which case the above reduces to
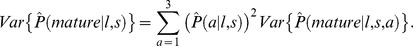
where the variance of 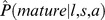 can be obtained using the model outputs in R. To account for uncertainty in 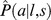 would require having estimates of uncertainty for the CPUE abundance estimates, the estimated length distributions, and the sex ratio estimates. Variance formulas for the product of random variables would then be required.

## Results

### Maturity classification and catch composition

**Figure 3:**
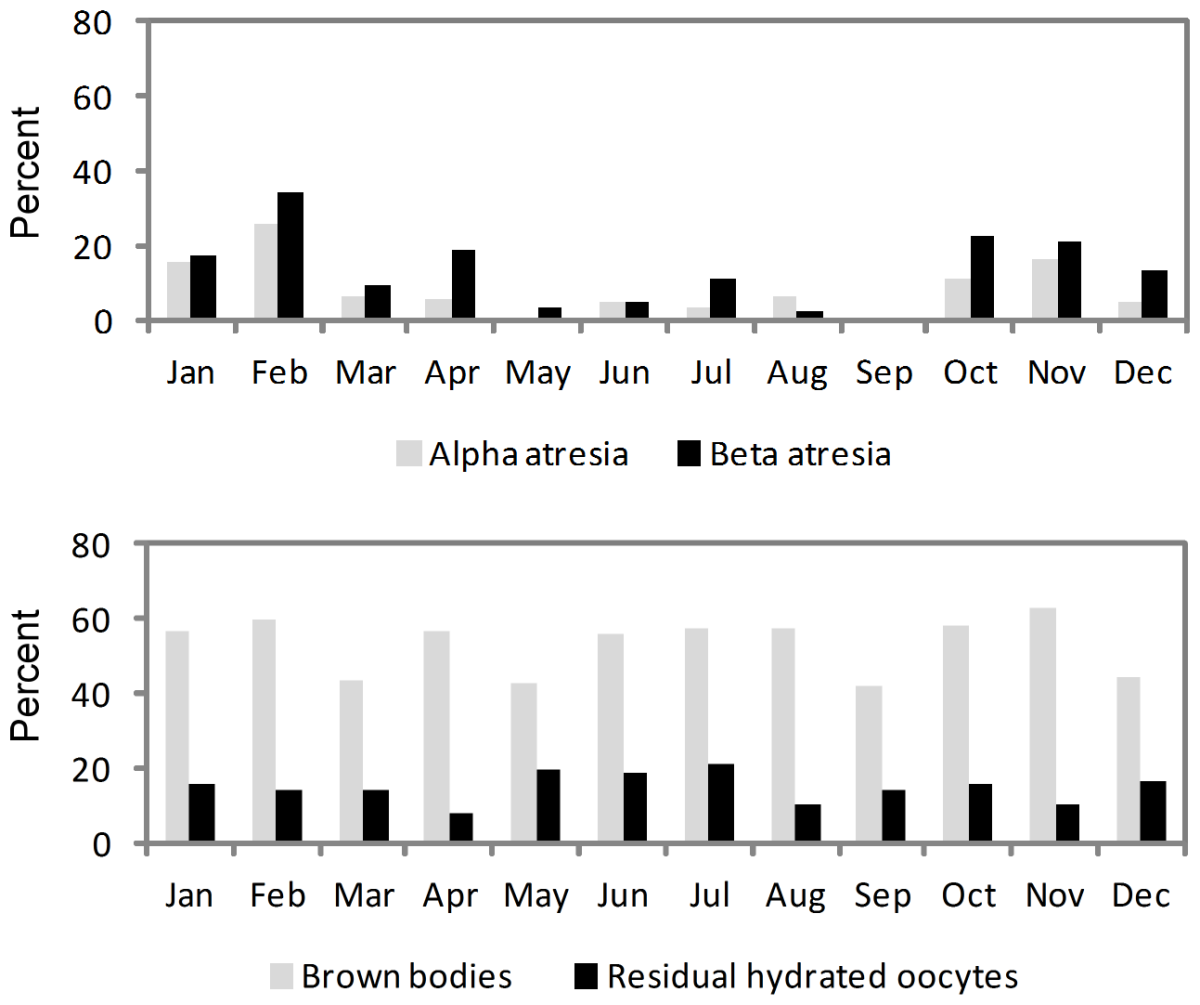
Percent of females ≥70 cm *FL* by month with alpha/beta atresia and maturity markers. Maturity markers are late stage atresia (gamma/delta; brown bodies) or residual (unovulated) hydrated oocytes.

Yolked oocytes in either the alpha or beta stages of atresia were found in 16.5% of ovaries of females ≥70 cm *FL*, while maturity markers were found in 56.3% of the ovaries. The percentage ovaries containing alpha and beta stage atresia was highest during the spawning season and lowest during the non-spawning period (Fig. 3), confirming that these stages of atresia do not persist in the ovary throughout the non-spawning months in albacore. By comparison, the percentage of ovaries containing brown bodies (gamma/delta atresia) and residual hydrated oocytes was relatively constant across months suggesting that these structures persist in ovaries until at least the following spawning season before being completely resorbed (Fig. 3). Gamma/delta atresia appeared as small structures scattered uniformly throughout the ovary section, and can be used as evidence of previous development of yolked oocytes and the attainment of maturity.

**Figure 4:**
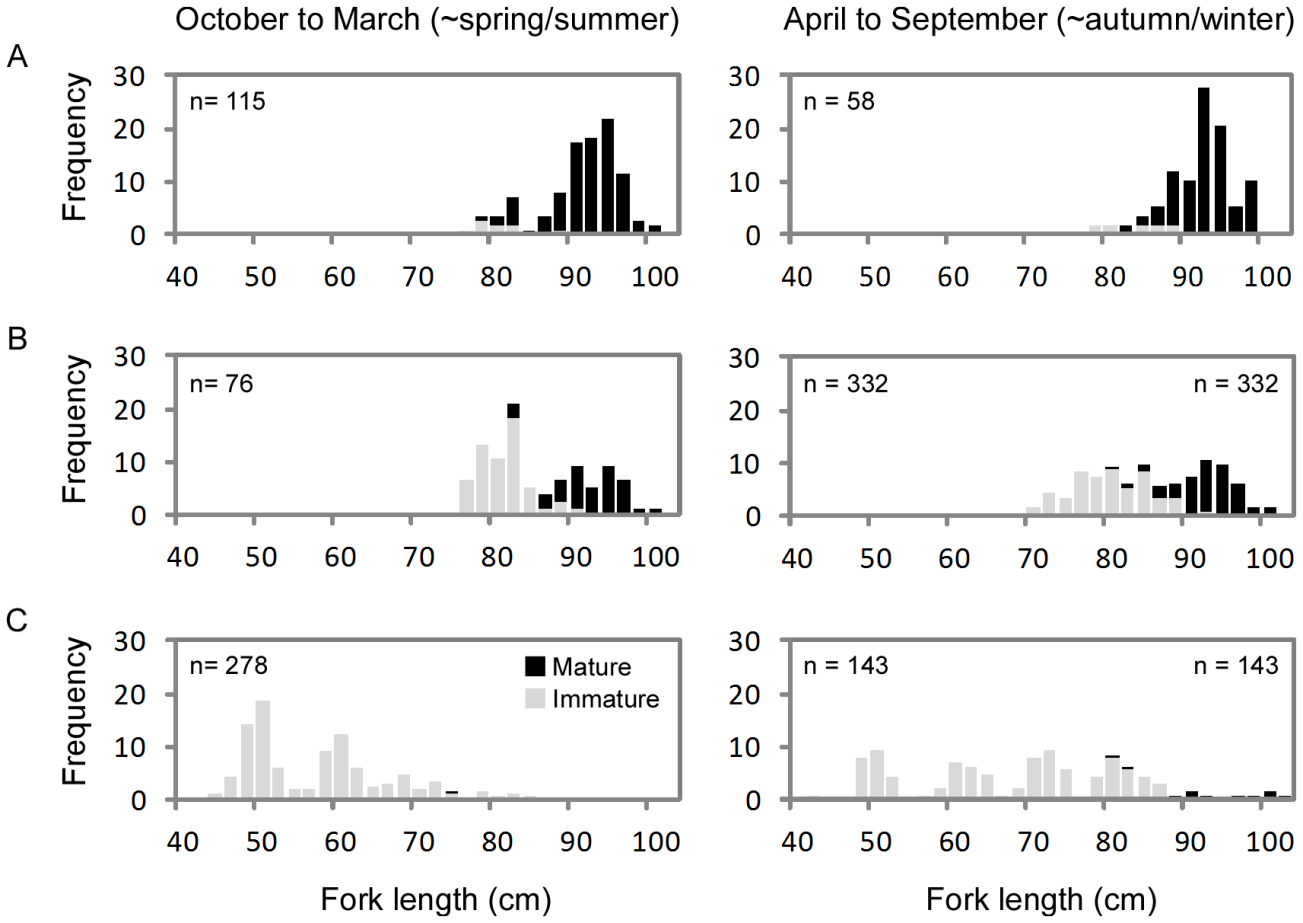
Length frequency (%) of immature and mature albacore sampled during spring/summer (left) and autumn/winter (right). The data were restricted to females sampled west of 175°E and are shown at three latitudes: (A) 14–20°S, (B) 20–30°S, (C) >30°S.

In total, 684 of the females sampled were classified as immature (43–94 cm *FL*) and 812 as mature (74–109 cm *FL*) (Table 1). Importantly, 843 of the fish sampled were from the transitional size range from 75 to 95 cm *FL*. Immature females made up a small proportion of the females sampled north of 20°S, while mature fish made up a small proportion of the females sampled south of 30°S (Fig. 1). Preliminary data exploration in the area west of 175°E (for which samples were collected across a wide range of latitudes), clearly shows different geographic distributions of mature and immature females during the year (Fig. 4). In the main spawning latitudes of 14–20°S, over 90% of fish sampled in the two seasons examined (spring/summer and autumn/winter) were mature (Fig. 4A). As latitude increased (became more southerly), the proportion mature decreased; by latitude >30°S, less than 4% of females sampled were mature (Fig. 4C). More importantly, the data showed that the proportion mature at length varied with latitude. For example, of the females 80–90 cm *FL* sampled during spring/summer, 80.8% were mature at 14–20°S compared to only 19.4% at 20–30°S, and 0.0% at >30°S. This is because fish in southern latitudes that are mature will move north to spawn during the spring/summer spawning season, leaving behind those fish of the same size that are immature. Although mature females were sampled at the most northerly (spawning) latitudes year-round, the presence of mature fish at 20–30°S and >30°S in autumn/winter (Fig. 4) suggests that some mature fish migrate south after spawning.

**Figure 5:**
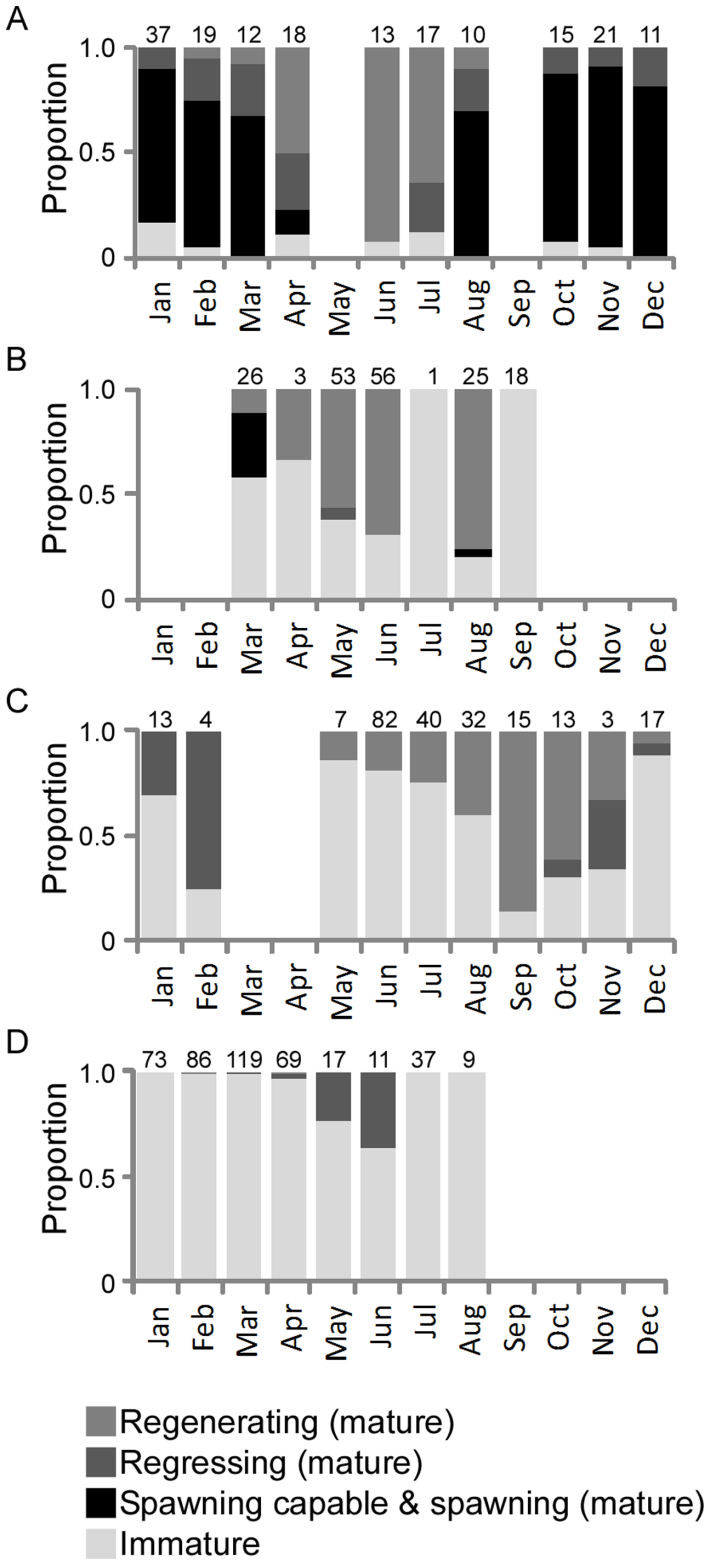
Proportion of females by development class, month and latitude west of 175°E. (A) 14–20°S, (B) 20–25°S, (C) 25–30°S, (D) >30°S. The regressing class includes classes 5, 6a and 6b (see Table 1). Sample size per month shown at top.

As expected, active females (spawning capable and spawning) dominated the sampling between October and March north of 20°S (Fig. 5A). At these latitudes, the relative abundance of females classed as mature-inactive (regressing and then regenerating) increased steadily from the middle of the spawning season (∼January) until June as individuals completed spawning. At 20–25°S, the relative abundance of regenerating females increased between March and August, which is slightly later than observed to the north (Fig. 5B). At 25–30°S, regenerating females increased in relative abundance even later in the year, between May and September, and then decreased, presumably because the mature females migrated north again to spawn the following spring/summer (Fig. 5C). Although rarely sampled, the presence of regenerating females south of 30°S in winter confirms that at least some mature fish migrate south after spawning (Fig. 5D).

### Spatial and temporal variation in maturity

**Figure 6:**
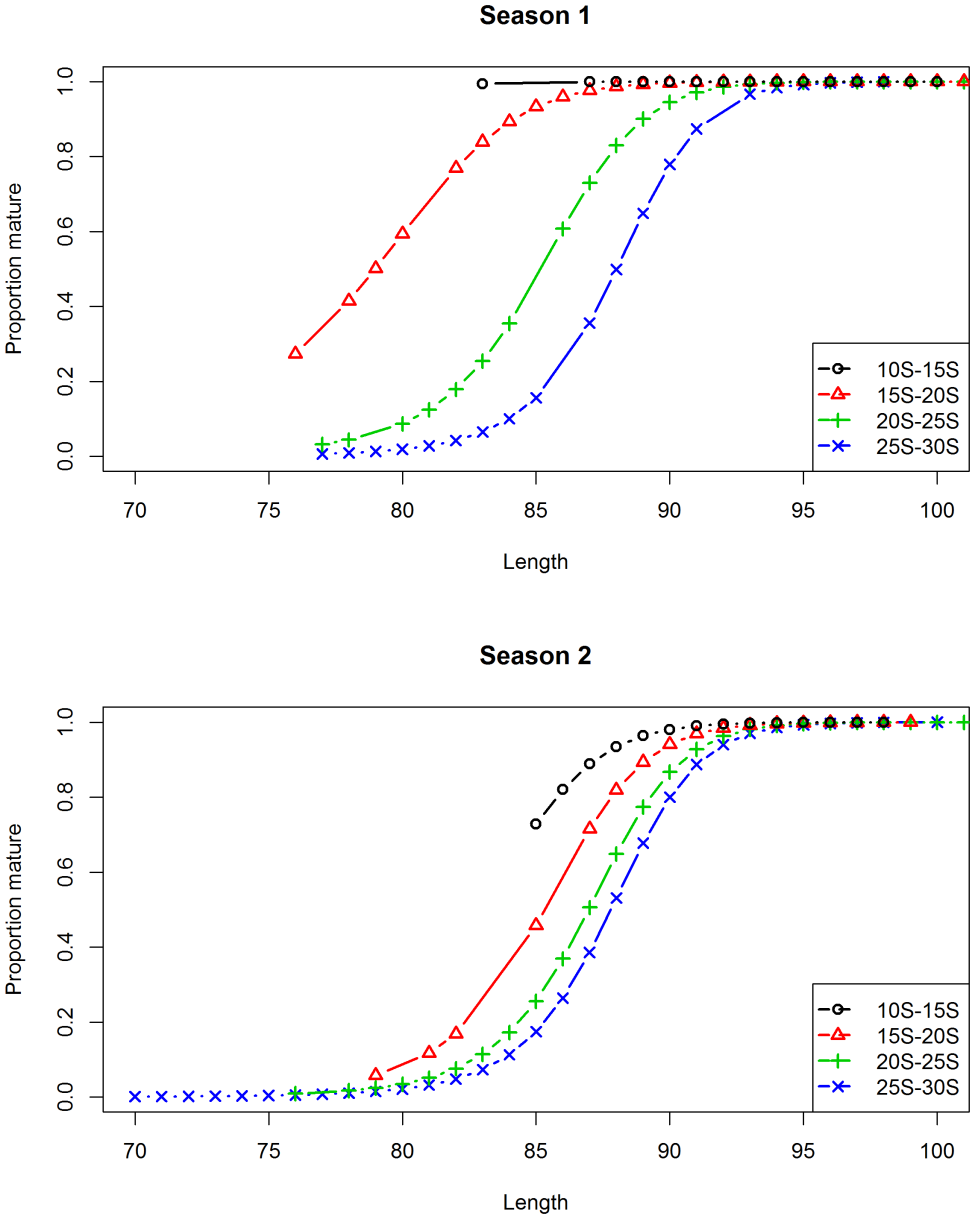
Predicted proportion of mature females by fork length and season at four latitudes. Season 1 = October to March, Season 2 = April to September.

**Figure 7:**
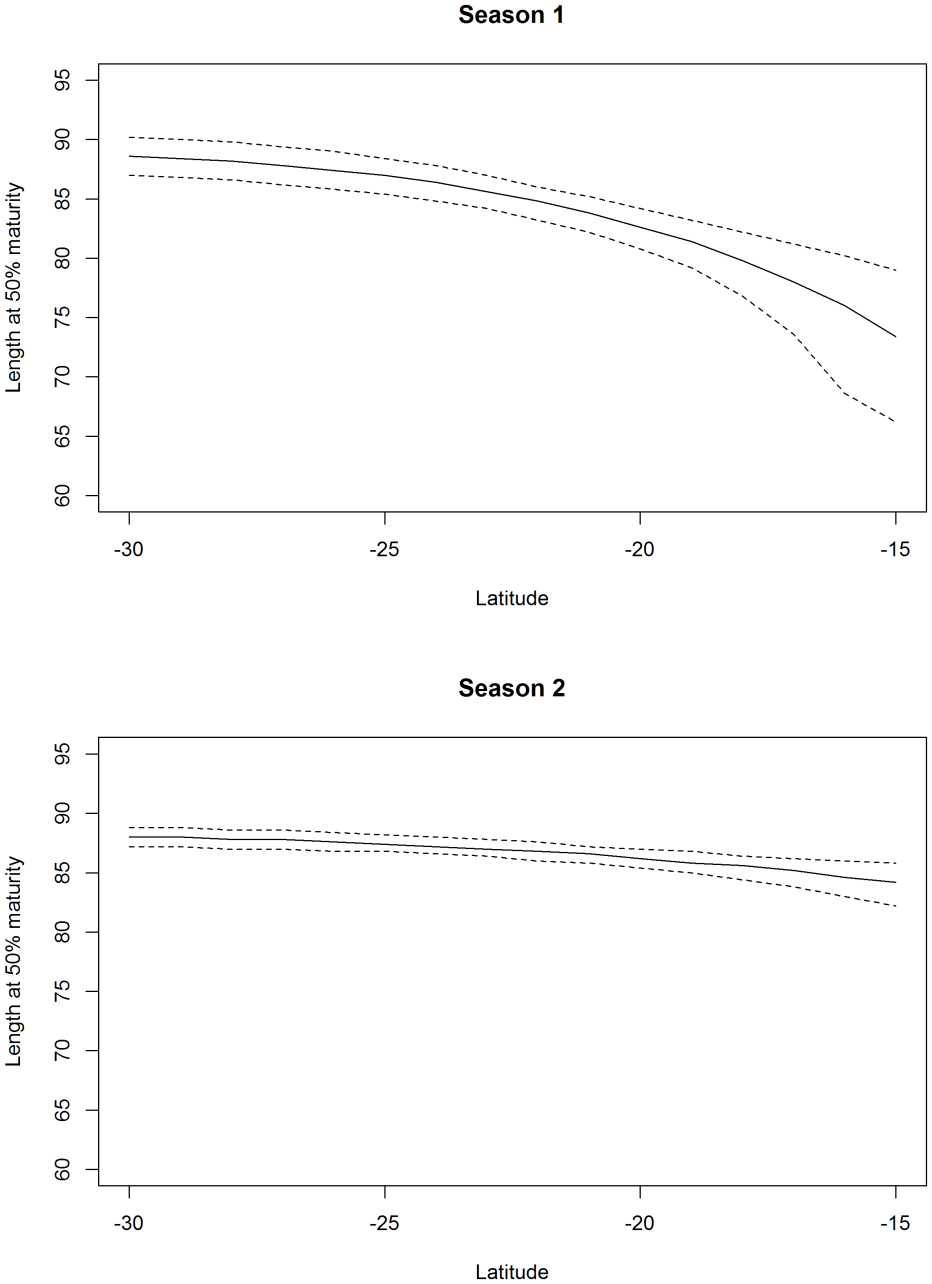
Predicted fork length at 50% maturity by latitude and season. Dashed lines represent 95% confidence intervals. Season 1 = October to March, Season 2 = April to September.

Six of the candidate models for describing the relationship between maturity and length had AICc weights greater than 1% (Table 2). The model that included covariates for latitude and two seasons, but not longitude, and an interaction between latitude and season, gave the best fit to the data (Table 2, Model 1, AICc weight 46.1%). Based on this model, females were predicted to be mature at significantly smaller lengths closer to the equator and between October and March (season 1), roughly corresponding to the Austral spring and summer (Fig. 6). The predicted pattern in proportion mature at length was similar at latitudes 30°S and 40°S where the length at 50% maturity was largest. The pattern in maturity at length was intermediate at 20°S (Fig. 6). The estimated length at 50% maturity was approximately 88 cm *FL* between April and September between latitudes 25–30°S, and decreased significantly in the more northerly latitudes during the warmer months from October to March, reaching approximately 70–80 cm *FL* at 15°S (Fig. 7). There was also some support for including longitude in the model (Models 2, 3 and 6 with AICc weights of 33.2%, 12.4%, and 1.3%), suggesting a moderately higher rate of maturity at length further east. There was limited support for quarterly variation in maturity (Models 5 and 6 with AICc weights of 2.2% and 1.3%).

In five of the six best fitting models, length was fitted as a cubic spline with 2 df (Table 2). The remaining model (Table 2, Model 4) included length as a linear covariate (i.e., df = 1), which corresponds to the customary logistic model; however, it had relatively limited support with an AICc weight of 3.3%.

Only one candidate model describing the relationship between maturity and age had an AIC_c_ weight greater than 1% (Table 3). Similar to maturity at length, the best-fitting model included a covariate for latitude, but not longitude, and an interaction between latitude and season (quarterly seasons in this case) (Table 3, Model 1). Based on a comparison of the best approximating models for length and age, age was a far worse predictor of maturity than length (Table 4).

**Table 3:** Comparison of the best 3 age-based models.

Model	Age df	Lat df	Ssn	Lat*Ssn	Δ AIC_c_	AIC_c_ weights
1	6	2	4	Y	0	100%
2	2	2	4	Y	20.84	0.0%
3	6	2	2	Y	23.42	0.0%

The Age df, Lat df, and Lon df columns specify the number of degrees of freedom (df) in the cubic spline used to define the relationship of the parameters length, latitude, and longitude with maturity. Where no df are given, the parameter was not included in the model. The Ssn column specifies the number of levels included in the model for season, either 4 (Jan–Mar, Apr–Jun, Jul–Sep, Oct–Dec) or 2 (Oct–Mar, Apr–Sep). A “Y” in the column for Lat*Ssn indicates that an interaction term between latitude and season was included.

**Table 4:** Comparison of the best-fitting age-based model with the equivalent length-based model given the same dataset.

Parameter	AIC_c_	Δ AIC_c_	AIC_c_ weights
Age	354.98	82.46	1.24E-18
Length	272.52	0.000	1.000

Both models include a cubic spline with 6 degrees of freedom for age or length, a cubic spline with 2 degrees of freedom for latitude, a categorical variable with 4 levels for season, and an interaction between the latitude and season terms.

### Maturity ogive estimation

**Figure 8:**
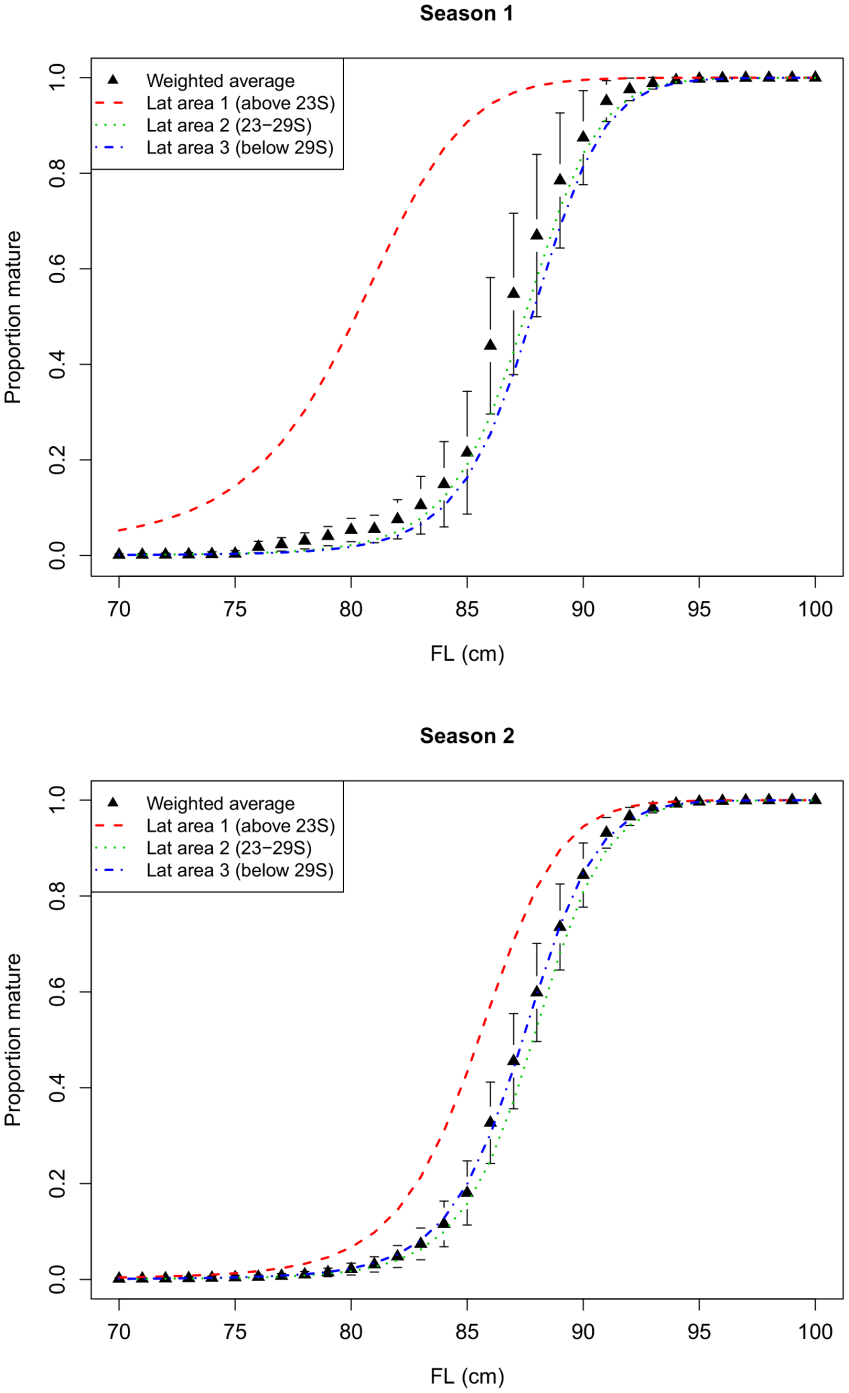
Predicted proportion of mature females by length and season. Coloured lines show the results for each latitudinal (lat) area of the ETBF. Black points show the weighted average across all regions, with error bars giving approximate 95% CIs. Note that the CIs are underestimated because they do not include uncertainty in the weights (i.e., in the estimates of relative abundance of females by length in each area).

The estimated maturity ogives from the model where latitude was included as a discrete factor show that a higher proportion of females in the northern-most latitudinal area (<23°S) are mature at a given length than in the areas further south, and this is more pronounced in season 1 than season 2 (Fig. 8). These results are consistent with results from the model where latitude was included as a continuous spline. Although the mean maturity at length for females in latitudinal area 2 was less than for area 3 (Fig. 8), the maturity curves for these two areas were not significantly different when confidence intervals were accounted for.

**Figure 9:**
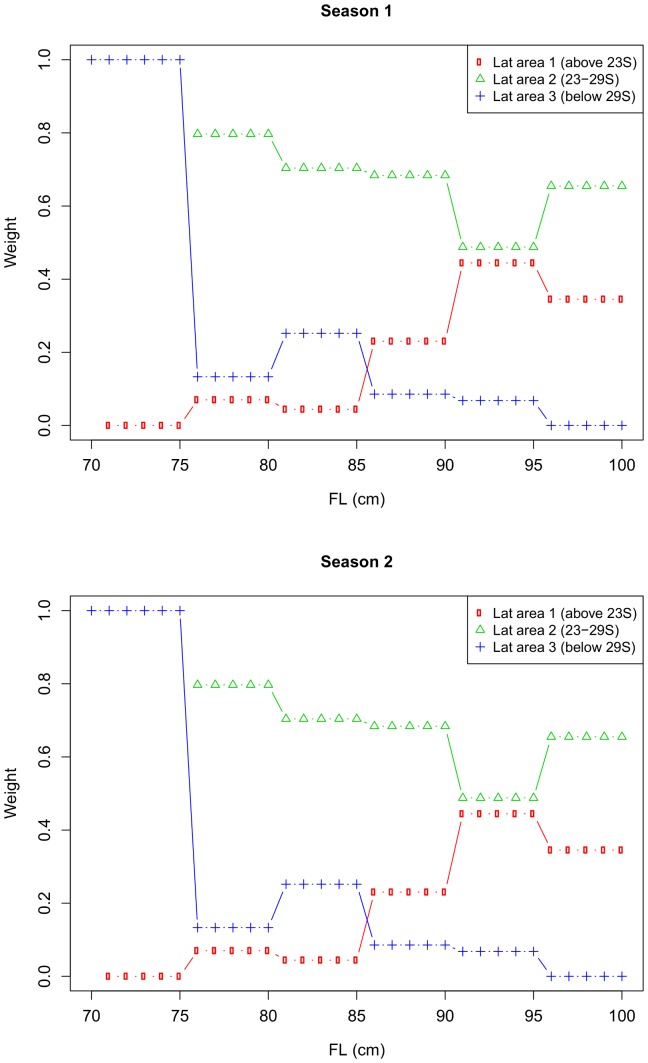
Weights given to the predicted proportion of mature females by latitudinal (lat) areas and season. The weights are the estimated proportion of females in a given 5-cm length class found in each of the three areas in a given season.

The weight assigned to the predicted proportion mature from each area (i.e., the relative abundance of females in each area) varied by length and season (Fig. 9). Using these weights, we calculated the predicted proportion of mature females by length for the whole ETBF in each season (Fig. 8). In both seasons, the estimated length at 50% maturity for females in the ETBF was approximately 87 cm *FL*, and length at 100% maturity was approximately 94 cm *FL*. Using the predicted growth curve for female albacore at 150°E [21], an 87 cm *FL* female would be 4.5 years old, and a 94 cm *FL* female would be 7 years old. The standard errors for the weighted maturity ogive (Fig. 8) were underestimated because they do not contain any uncertainty in the weights, i.e., in the estimates of relative abundance, length distribution and sex ratio. If we had uncertainty estimates for these components, they could be incorporated into the estimated standard errors.

**Figure 10:**
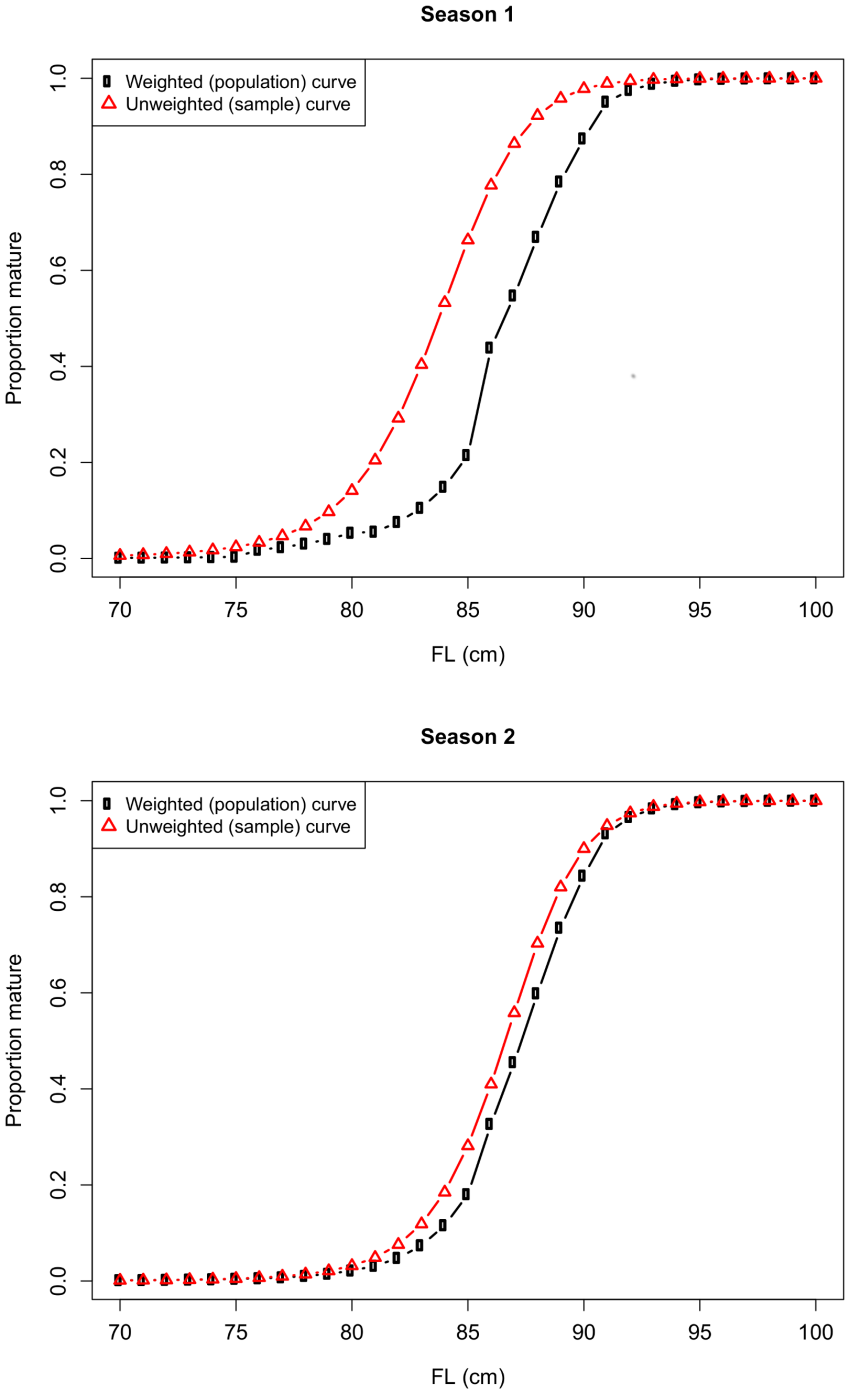
Comparison of the weighted and unweighted maturity ogives for albacore by season. In contrast to the unweighted ogives, the weighted ogives take latitudinal variation in maturity and relative abundance into account and should be representative of the female population for the whole Eastern Tuna and Billfish Fishery (ETBF).

For comparison, we also calculated an “unweighted” maturity ogive for each season in the ETBF by fitting a logistic model to the data without considering latitudinal differences (i.e., without a covariate for latitude). The weighted and unweighted curves were similar in season 2, but not in season 1 (Fig. 10). The estimated length at 50% maturity in season 1 was almost 3 cm less (84 cm versus 87 cm) when latitudinal differences in maturity and relative abundance were not taken into account. In season 1, samples were primarily taken from the spawning ground (northern-most latitude band) and under-represented immature fish, so the unweighted maturity ogive was biased low for the population.

## Discussion

The data in this study were collected using consistent histological criteria to classify the maturity status of females caught across a broad area of the South Pacific Ocean. The criteria were precise enough to distinguish mature but regenerating females from immature females well after the spawning season by the presence of maturity markers, such as encapsulated hydrated oocytes and/or brown bodies (gamma/delta atresia). Although brown bodies have been identified in bluefin tuna ovaries (called yellow pigment; [22], [23]), they have not been used to distinguish between mature-regenerating and immature females in tuna. Gamma and delta atresia are, however, used to identify mature-regenerating females in many other fish species [14], [24], [25], [26] and are a histological feature of regenerating females listed in a recent “standardized universal terminology for the phases in the reproductive cycle of fish” [14].

For many fish, mature and immature females can only be differentiated if sampling occurs prior to the spawning season when mature regenerating females are uncommon [27]. At other times of the year, the ovaries of mature but regenerating females appear immature because all postovulatory follicles and yolked oocytes are resorbed. The criteria used in the current study, especially the late stages of atresia; however, appear to be appropriate for identifying mature albacore throughout the reproductive cycle. Three lines of evidence support this conclusion. First, the proportion of females with maturity markers did not decline at any stage during the year, unlike the proportion with alpha or beta atresia, which declined after the spawning season. This demonstrates that resorption of maturity markers is a slow and protracted process. Second, the size range at which females attained sexual maturity in our study was relatively narrow (74–94 cm *FL*; 20 cm) and the largest female classified as immature was 94 cm *FL*, which is the same as found in a recent study of North Pacific albacore [28]. The absence of very large ‘immature’ females again suggests that the classification scheme is correctly identifying mature regenerating females. Finally, the distribution and seasonal change in the relative abundance of development classes is consistent with the assumed seasonal distribution and movement patterns of albacore in the South Pacific. The results clearly show that in the southwest Pacific, immature females make up a small proportion of the population in the spawning latitudes and become progressively more dominant to the south. The seasonal change in the relative abundances of mature regressing and regenerating females with latitude suggests a migration pattern where a proportion of post-spawning females migrate south after spawning, arriving at higher latitudes progressively later in the year. Their relative abundance at high latitudes then declines prior to the following spawning season as the regenerating females move north again to develop their gonads and spawn. This pattern of movement with latitude is consistent with catch rate data for albacore in the ETBF [20] and the broader western and central Pacific [29]. Seasonal migration between the tropics and sub-tropics has been inferred from peaks in longline catches in the subtropics in December to January and May to July, and is thought to be linked to the seasonal movements of specific temperature isotherms [29], [30], [31].

Our modelling results confirm that the proportion mature at length for female albacore varies with both latitude and season in the southwest Pacific and that this variation can be attributed to different geographic distributions and migration of mature and immature fish across the year. There was also some support for higher probability of maturity at length further east. Such longitudinal effects are also seen in albacore growth, with faster growth rates and higher asymptotic lengths further east [21]. However, almost all samples from the east (>180°E) were large mature fish collected from the northern spawning latitudes, so data for comparing maturity ogives by longitude were limited.

Length was much more closely associated with the probability of females being mature than was age, which is consistent with the process of maturation for individual fish being driven by fish size (among other factors such as condition) rather than age. However, length measurement is more precise than age estimation which may contribute to the tighter relationship. Although South Pacific albacore growth rates vary with longitude, with greater length-at-age further east [21], we found little support for a relationship between longitude and maturity at age. Given the moderate support for a relationship between longitude and maturity at length, maturity might be expected at younger ages in the east. At an individual level, such a relationship was not detected, though this may be due to a lack of statistical power, either because maturity is driven mostly by length and variable growth rates added too much statistical noise, or because of ageing uncertainty.

Logistic curves are often used to model maturation, but in this case the more flexible cubic splines provided a better fit to the data. The pattern of maturation appeared to be asymmetrical across sizes, with the rate of accumulation of mature fish increasing with size. The size at 50% maturity of albacore was quite close to the asymptotic length for females (95.5 cm *FL* in the west; [21]) and growth slows quite rapidly as they approach the asymptotic length. An asymmetric pattern of maturation has also been found in yellowfin (*Thunnus albacares*) [32] and bigeye (*T. obesus*) [33] tuna.

Spatial variability in maturity at length/age clearly must be accounted for when estimating a single population maturity schedule. In order to obtain a maturity ogive that is representative of the population, samples need to be taken from the stock's entire geographic distribution and weighted by relative population abundance across the distribution. When the seasonal movements differ between mature and immature fish, it is also important to take seasonal variability into account. This was the case for albacore in the South Pacific, since mature fish tended to move to the northern spawning latitudes during the spawning season. As such, we calculated separate population maturity ogives for the spawning and non-spawning seasons. In theory, weighted maturity ogives derived from different seasons would be the same (except for sampling variability) for a closed population, because the same population of fish would be sampled in both seasons. The maturity ogives we derived for albacore in the ETBF region were reasonably similar; however, we would not necessarily expect them to be the same since the ETBF does not represent a closed population (i.e., albacore can move in and out of the ETBF).

The method proposed here to obtain a single ogive by weighting the maturity at length data by an index of female abundance within latitude bands is appropriate for albacore. Similar methods to account for spatial variation in maturity at length in other species have been suggested [8], [34]. An index of abundance by latitude and sex is not available for albacore across the South Pacific, but is available for Australia's ETBF region, so the maturity ogives we calculated here are only representative of the ETBF population. Furthermore, the method requires information on the size distribution of albacore within each latitudinal area. Unbiased size data for albacore catches in the ETBF are currently not available [35]. Using the length data from fish sampled in [6] allowed us to obtain a preliminary estimate of the maturity ogive for albacore in the Australian region. The preliminary estimate of length at 50% maturity of 87 cm *FL* is within the range obtained previously [28], [36], [37], [38]. However, the predicted age at 50% maturity of 4.5 years and 100% maturity of 7 years is different to that currently assumed in stock assessments in the north Pacific, Atlantic and Indian Oceans which all assume that 50% maturity is reached at age 5 and full maturity at age 6 [12], [39], [40], [41]. In the Mediterranean, 50% maturity is estimated at 66 cm *FL* and age two or three years depending on the growth curve used [42].

The observed patterns in maturity have implications for future stock assessments of South Pacific albacore. Perhaps most importantly, our results provide a better understanding of the population structure, which can be used to inform alternative assessment model structures. Changes in assessment model structure can have far more effect on stock status estimates than changing a single parameter estimate [11]. For example, the migration of South Pacific albacore according to maturity and season may require structural modifications to the current assessment such as age-dependent seasonal movement between separate regions. The possible trends in length at maturity with longitude, in parallel with the longitudinal growth variation, further suggest the potential to modify the regional structure of the stock assessment. Such structural refinements are likely to provide more reliable estimates of reproductive potential, spawning stock biomass, and potential yields, and ultimately provide an improved foundation for management decisions.
